# Leptin Receptor Blockade Attenuates Hypertension, but Does Not Affect Ventilatory Response to Hypoxia in a Model of Polygenic Obesity

**DOI:** 10.3389/fphys.2021.688375

**Published:** 2021-07-02

**Authors:** Lenise J. Kim, Mi-Kyung Shin, Huy Pho, Laszlo Otvos, Sergio Tufik, Monica L. Andersen, Luu V. Pham, Vsevolod Y. Polotsky

**Affiliations:** ^1^Division of Pulmonary and Critical Care Medicine, Department of Medicine, Johns Hopkins University School of Medicine, Baltimore, MD, United States; ^2^Institute of Medical Microbiology, Semmelweis University, Budapest, Hungary; ^3^Arrevus, Inc., Raleigh, NC, United States; ^4^OLPE, LLC, Audubon, PA, United States; ^5^Department of Psychobiology, Universidade Federal de São Paulo, São Paulo, Brazil

**Keywords:** leptin, obesity, blood pressure, hypoxic ventilatory response, sleep-disordered breathing, leptin receptor blocker

## Abstract

**Background:**

Obesity can cause hypertension and exacerbates sleep-disordered breathing (SDB). Leptin is an adipocyte-produced hormone, which increases metabolic rate, suppresses appetite, modulates control of breathing, and increases blood pressure. Obese individuals with high circulating levels of leptin are resistant to metabolic and respiratory effects of leptin, but they appear to be sensitive to hypertensive effects of this hormone. Obesity-induced hypertension has been associated with hyperleptinemia. New Zealand obese (NZO) mice, a model of polygenic obesity, have high levels of circulating leptin and hypertension, and are prone to develop SDB, similarly to human obesity. We hypothesize that systemic leptin receptor blocker Allo-aca will treat hypertension in NZO mice without any effect on body weight, food intake, or breathing.

**Methods:**

Male NZO mice, 12–13 weeks of age, were treated with Allo-aca (*n* = 6) or a control peptide Gly11 (*n* = 12) for 8 consecutive days. Doses of 0.2 mg/kg were administered subcutaneously 2×/day, at 10 AM and 6 PM. Blood pressure was measured by telemetry for 48 h before and during peptide infusion. Ventilation was assessed by whole-body barometric plethysmography, control of breathing was examined by assessing the hypoxic ventilatory response (HVR), and polysomnography was performed during light-phase at baseline and during treatment. Heart rate variability analyses were performed to estimate the cardiac autonomic balance.

**Results:**

Systemic leptin receptor blockade with Allo-aca did not affect body weight, body temperature, and food intake in NZO mice. Plasma levels of leptin did not change after the treatment with either Allo-aca or the control peptide Gy11. NZO mice were hypertensive at baseline and leptin receptor blocker Allo-aca significantly reduced the mean arterial pressure from 134.9 ± 3.1 to 124.9 ± 5.7 mmHg during the light phase (*P* < 0.05), whereas the control peptide had no effect. Leptin receptor blockade did not change the heart rate or cardiac autonomic balance. Allo-aca did not affect minute ventilation under normoxic or hypoxic conditions and HVR. Ventilation, apnea index, and oxygen desaturation during NREM and REM sleep did not change with leptin receptor blockade.

**Conclusion:**

Systemic leptin receptor blockade attenuates hypertension in NZO mice, but does not exacerbate obesity and SDB. Thus, leptin receptor blockade represents a potential pharmacotherapy for obesity-associated hypertension.

## Introduction

Obesity is a major public health problem affecting 35% of United States adults (Flegal et al., 2010). Obesity increases cardiovascular morbidity and mortality (Hubert et al., 1983; Hall et al., 2001; Rahmouni et al., 2005a; Adams et al., 2006). Hypertension is observed in 74% of obese individuals and the risk of resistant hypertension is fivefold higher in obesity compared to normal weight patients (Bramlage et al., 2004). Obesity also causes sleep-disordered breathing (SDB), which contributes to hypertension (Peppard et al., 2000; Young et al., 2002; Wolk et al., 2003). Multiple mechanisms may be involved in the pathogenesis of obesity-induced hypertension, one of which is related to high circulating levels of leptin (Rausch et al., 1991; Hall et al., 2001, 2010; Rahmouni et al., 2005a,b; Friedman, 2009; Bell and Rahmouni, 2016).

Leptin, an adipocyte-produced hormone, plays an important role in obesity and its sequelae. Leptin exerts beneficial effects on metabolism by suppressing appetite and increasing the metabolic rate (Halaas et al., 1995; Spiegelman and Flier, 2001; Friedman, 2009), mitigating obesity. Leptin has a profound effect on control of breathing, stimulating ventilation (O’Donnell et al., 1999; Pho et al., 2016, 2021; Yao et al., 2016) and mitigating SDB. On the other hand, leptin activates the sympathetic nervous system and increases blood pressure (Asferg et al., 2010; Shankar and Xiao, 2010; Kshatriya et al., 2011; Bell and Rahmouni, 2016; Shin et al., 2019a, 2021), predisposing to hypertension. Obese individuals are resistant to the metabolic and respiratory effects of leptin, but are sensitive to the hypertensive effects of this hormone (Maffei et al., 1995; Considine et al., 1996; Ip et al., 2000; Phipps et al., 2002; Rahmouni et al., 2005b; Yee et al., 2006; Simonds et al., 2014; Fleury Curado et al., 2018; Berger et al., 2019). Although precise molecular mechanisms of this dichotomy remain poorly understood, limited permeability of the blood-brain barrier (BBB) to leptin could be involved in the process (Schwartz et al., 1996; Banks et al., 1999; Morris and Rui, 2009; Scarpace and Zhang, 2009; Wauman and Tavernier, 2011). Under these circumstances, the blockade of leptin signaling in leptin resistant obesity would treat hypertension, but would not affect metabolism or respiratory control. Here, we propose to address this hypothesis using an animal model of obesity-induced hypertension and hyperleptinemia, which mimics human obesity.

New Zealand obese (NZO) mice are an inbred strain representing polygenic-spontaneous obesity (Bielschowsky and Goodall, 1970; Haskell et al., 2002; Joost and Schürmann, 2014). NZO mice naturally develop hypertension, insulin resistance, and glucose intolerance (Ortlepp et al., 2000; Koza et al., 2004; Joost and Schürmann, 2014). NZO mice have altered upper airway anatomy characterized by increased volume of pharyngeal soft tissues and fat deposits in the tongue (Brennick et al., 2009), which predispose to SDB (Hernandez et al., 2012; Baum et al., 2018). Hyperleptinemia and leptin resistance are also key traits of NZO mice (Halaas et al., 1995; Igel et al., 1997; Wator et al., 2008). However, it is still unknown whether leptin plays a role in the pathogenesis of obesity-induced hypertension and SDB in NZO mice. We used a systemic leptin receptor blocker, Allo-aca, to evaluate the effects of leptin on blood pressure and breathing of NZO mice. We hypothesized that systemic blockade of leptin signaling will treat hypertension in NZO mice without exacerbating obesity and SDB.

## Materials and Methods

### Animals

In total, 18 male NZO mice, 12–13 weeks of age, from Jackson Laboratory (Bar Harbor, ME, United States, Stock #002105) were used in our experiments. They were housed in individual cages with water and chow diet *ad libitum*. Animals were maintained at 22°C and at a 12 h-light/dark cycle with lights on at 9 AM. Body weight, body temperature, and food consumption were monitored throughout the experiments. The study was approved by the Johns Hopkins University Animal Care and Use Committee (ACUC) under the protocol number #MO18M211.

### Surgical Procedures

All the surgical procedures were performed under sterile, aseptic conditions. Animals were anesthetized using 1–2% isoflurane administered through a facemask. The adequacy of anesthesia was assessed by the breathing frequency and the absence of forelimb or hindlimb pedal withdrawal reflex. Body temperature was kept around 37°C with a heating pad during the surgery. Surgical sites were washed with betadyne scrub solution. Sterile ophthalmic ointment was used for lubrication of the eyes. Immediately after the surgeries, all mice received 0.05 mg/kg of Buprenorphine intraperitoneally and were housed in a recovery chamber under a heating lamp. Mice were monitored and received additional 0.05 mg/kg of Buprenorphine for the next 3 days or until no signs of distress or pain were observed.

Initially, the NZO mice underwent a telemetry implantation for blood pressure recording as previously described by our group (Jun et al., 2014; Shin et al., 2019a, 2021). Mice were implanted with PA-C10 telemeters (Data Sciences International, St. Paul, MN, United States) into the left femoral artery. Mice were allowed to recover for 7–10 days.

After mice fully recovered, they were implanted with EEG/EMG electrodes using an EEG/EMG headmount (Pinnacle Technology, Lawrence, KS, United States) as previously described (Hernandez et al., 2012; Pho et al., 2016, 2021; Yao et al., 2016; Fleury Curado et al., 2018; Berger et al., 2019). Briefly, animals were placed in a stereotaxic frame and a longitudinal midline incision was performed on their skull under aseptic conditions. The underlying fascia was gently removed and the headmount was glued above the bregma. Two pairs of silver electrodes were screwed to the headmount with silver conductive epoxy in frontal and parietal regions bilaterally. Insulated EMG leads were placed over the nuchal muscles. The incision was sutured with 6-0 silk suture and the area around the headmount was closed with dental acrylic. Mice recovered for at least 7 days prior to the baseline measurements.

### Treatment

Six NZO mice were treated with a designer leptin receptor antagonist, Allo-aca (0.2 mg/kg), which was administered 2×/day at 10 AM and 6 PM *via* subcutaneous injections for 8 consecutive days. Injections were performed during light-phase when the animals are more likely to sleep. Allo-aca is a 9-residue peptidomimetic (H-alloThr-Glu-Nva-Val-Ala-Leu-Ser-Arg-Aca-NH_2_) based on the C-terminal site III of leptin with a putative mechanism of action by binding to circulating leptin receptor (Peelman et al., 2004; Otvos et al., 2011a,b). To date, Allo-aca appears to be the most active leptin receptor antagonist within a wide pharmaceutically acceptable concentration range *in vitro* and effectively produces weight gain and decreases blood pressure *in vivo* (Otvos et al., 2008, 2011a,2011b, 2014; Huby et al., 2016; You et al., 2016). Allo-aca shows ObR antagonist properties at 0.1 and 1 mg/kg daily doses with little dose-dependence. Receptor binding data have shown that the Allo-aca-ObR interaction half-time is 2 h and the peptide deactivates the receptor for 6–8 h (Otvos et al., 2008, 2011a,2011b, 2014). Moreover, Allo-aca temporarily crosses the BBB, promoting a blockade of leptin receptors both in the central nervous system (CNS) and in the periphery (Otvos et al., 2011b). Another group of 12 NZO mice received a control peptide Gly11 at the same dose and according to the same administration protocol as the Allo-aca group. Gly11 [(H-Chex-Arg-Pro-Asp-Lys-Pro-Arg-Pro-Tyr-Leu-Gly-Arg-Pro-Arg-Pro-Pro-Arg-Pro-Val-Arg)_2_-Dab-NH_2_] is an antimicrobial peptide derivative that was successfully used as a negative control of leptin receptor blockade in previous reports (Otvos et al., 2011a, 2018).

### Blood Pressure Recordings

Blood pressure and heart rate were recorded at baseline and from day 5 to day 7 of treatment with either Allo-aca (*n* = 5) or Gly11 (*n* = 8). Recordings were performed by telemetry as previously described (Jun et al., 2014; Shin et al., 2019a, 2021). Signals were obtained and processed using PowerLabs 16/35 interfaced with LabChart 7 Pro software (version 7.2) from ADInstruments (Colorado Springs, CO, United States). Signals were sampled at 400 Hz per second. Systolic (SBP) and diastolic blood pressure (DBP) were averaged every 1 h and used to calculate the mean arterial pressure (MAP) during light and dark-phase.

### Heart Rate Variability Analyses

Heart rate variability was analyzed to estimate the sympathetic and parasympathetic activities, and the cardiac autonomic balance, as previously reported (Malliani et al., 1991; Zoccal et al., 2009; Moraes et al., 2014), at baseline and after the treatment with either Allo-aca (*n* = 4) or control peptide (*n* = 7). Frequency components of heart rate could not be extracted from two mice due to technical problems in the data processing. The heart rate recordings were sampled at 400 Hz. To remove artifacts, we excluded heart rate readings <200 or >800 bpm, and imputed values with linear interpolation. The reciprocal of the heart rate was then used to estimate the beat-to-beat interval. These recordings were then windowed into 2-min segments, which overlapped by 1 min, with a Hamming window. A fast Fourier transform was applied to each segment to calculate the power spectral density with a frequency resolution of 0.1 Hz. Low frequency power (LF) was defined as the sum of the power in the 0.4–1.5 Hz range, representing the cardiac sympathetic activity. High frequency power (HF) was defined as the sum of the power in the 1.6–4.0 Hz range, as a representative of cardiac parasympathetic activity. The ratio of the LF to HF power was also calculated (LF/HF) to estimate the cardiac autonomic balance. The data were further reduced by averaging each 2 min segment for each mouse in the baseline and treatment conditions. The analyses were performed in MATLAB software (The MathWorks, Natick, MA, United States).

### Hypoxic Ventilatory Response

The hypoxic ventilatory response (HVR) was measured at baseline and at day 7 of treatment with either Allo-aca (*n* = 6) or Gly11 (*n* = 8) as previously described (Caballero-Eraso et al., 2019; Shin et al., 2019b). Briefly, HVR measurements were performed in a whole-body barometric plethysmography (WBP) chamber (Buxco, Wilmington, NC, United States) combined with a pulse oximetry system (STARR Life Sciences Corp., PA, United States) (Hernandez et al., 2012; Pho et al., 2016). All mice were acclimated to the WBP chamber and to the pulse oximeter collar for at least 3 days before the baseline recording. HVR was measured during quiet wakefulness. Animals were exposed to 2–3 cycles of normoxia/hypoxia per day separated by at least 30 min during the light-phase (between 10 AM and 5 PM). All measurements occurred under thermoneutral conditions (∼30°C) in a neonatal incubator (Ohio Medical, IL, United States). Ventilation was recorded under normoxic conditions (at 20.9% FiO_2_ and 0.4% CO_2_) over 20 min followed by 5 min of hypoxia in which the animals were exposed to a gas mixture of 10% O_2_ + 3% CO_2_ balanced in N_2_. We have previously shown that a fixed 3% CO_2_ tension circumvents the hyperventilation-induced hypocapnia during poikilocapnic hypoxia, maintaining similar partial pressure of CO_2_ (PaCO_2_) at normoxic and hypoxic conditions in mice (Caballero-Eraso et al., 2019). The FiO_2_ was decreased rapidly to 10% within the first 30 s. Minute ventilation (V_E_) and oxyhemoglobin saturation (SpO_2_) during hypoxia were measured based on a 90 s period (from the first 30 s of hypoxia exposition until 2 min). The first 2 min of acute hypoxia are characterized by an augmented ventilation mainly governed by peripheral chemoreflex (Duffin, 2007; Powell, 2007; Teppema and Dahan, 2010). HVR was calculated by the slope of the relationship between V_E_ and SpO_2_ at normoxic and hypoxic conditions *via* a linear least-squares regression analysis as previously described (Polotsky et al., 2004; Caballero-Eraso et al., 2019; Shin et al., 2019b). Both V_E_ in normoxia and hypoxia, and HVR were normalized by body weight. All signals were sampled at 1000 Hz (sampling frequency per channel) and recorded in LabChart 7 Pro (version 7.2).

### Sleep Studies

Sleep architecture and ventilation during sleep were examined by full-polysomnography using a WBP chamber at baseline and at day 8 of treatment with either Allo-aca (*n* = 6) or Gly11 (*n* = 7) as previously published by our group (Hernandez et al., 2012; Pho et al., 2016, 2021; Yao et al., 2016; Fleury Curado et al., 2018; Berger et al., 2019). All mice were acclimated to the WBP chamber and to the SpO_2_ collar as described above Sleep studies were conducted under constant temperature of ∼29°C and ∼90% humidity. Mice were recorded for 6 h from 10 AM to 4 PM. Signals were processed at 1000 Hz (sampling frequency per channel) and recorded in LabChart 7 Pro (version 7.2). Sleep was visually scored in 10-s epochs based on EEG and EMG activity as previously described (Hernandez et al., 2012; Pho et al., 2016, 2021; Yao et al., 2016; Fleury Curado et al., 2018; Berger et al., 2019). Epochs with high EEG amplitude in the low-frequency band (∼2–5 Hz) and low EMG tonus were classified as NREM sleep. Epochs with low-amplitude and mixed frequencies in the EEG (∼5–10 Hz) and muscle atonia were scored as REM sleep. Ventilation was analyzed during all REM sleep epochs (∼22 min in 6-h recording; see Table 1) and in 20-s subsamples from NREM sleep. Each NREM subsample was manually selected once per 30 min of recording. Each breath was further analyzed to determine the maximal inspiratory airflow (V_I_max), tidal volume (V_T_), respiratory rate (RR), mean inspiratory flow rate (MIFR), and inspiratory duty cycle (DC). V_E_ was normalized to body weight. Inspiratory flow limitation (IFL) was defined as an early inspiratory plateau in airflow associated with increasing respiratory effort as previously reported by our group (Hernandez et al., 2012; Pho et al., 2016, 2021). Apneas were defined as reductions of ≥90% in airflow during sleep for at least two breath cycles or ≥0.7 s (Freire et al., 2020). Apnea index was calculated by dividing the number of apneas by the total sleep time. Mean SpO_2_ was calculated during both NREM and REM sleep. Oxygen desaturation index (ODI) was defined as the number of ≥5% oxyhemoglobin desaturations from baseline divided by the total sleep time as previously described (Yao et al., 2016).

**TABLE 1:** Sleep architecture in NZO mice at baseline and after the treatment with leptin receptor blocker Allo-aca or control peptide Gly11.

Group	Time	*N*	Total sleep time (min)	NREM (%)	REM (%)	Sleep efficiency (%)
Gly11	Baseline	7	195.1 ± 16.1	88.5 ± 1.8	11.5 ± 1.8	60.1 ± 4.2
	Treatment	7	206.5 ± 17.9	89.3 ± 1.6	10.7 ± 1.6	60.1 ± 2.9
Allo-aca	Baseline	6	233.2 ± 15.7	91.9 ± 0.7	8.1 ± 0.7	63.8 ± 3.5
	Treatment	6	217.8 ± 17.7	92.3 ± 0.3	7.7 ± 0.3	61.5 ± 3.9

*Mean values ± standard errors are shown.*

### Plasma Leptin Levels

Blood was collected from mice at baseline and on the last day of treatment of either Allo-aca (*n* = 6) or Gly11 (*n* = 7). Blood collection was performed immediately after the sleep studies, while the mice were food and water deprived for 6 h. Blood samples were centrifuged at 3500 rpm for 15 min at 4°C. Leptin levels were measured in the plasma using a mouse leptin ELISA kit (#EZML82K, Millipore, MA, United States).

### Statistical Analysis

The main outcomes were MAP, HVR, and V_E_ during sleep. Blood pressure analyses were stratified by light vs. dark phase to account for diurnal variations. For each outcome, mixed effects linear regression models were developed to test the independent effects of Allo-aca or Gly11 treatments and evaluate the differences within-subjects compared to baseline. The goodness-of-fit of each model was assessed by verifying the normality of the residuals. Considering the reduced sample size, the comparisons from mixed effects regression models were also performed with additional non-parametric tests as a confirmatory method. Mann–Whitney U test and Wilcoxon signed-rank test were used to analyze differences between the groups and within-subjects, respectively. *Post hoc* power analysis was performed considering the mean differences in MAP between groups. Given our sample size, we had the ability to detect 9 mmHg difference in MAP between treated and control animals (Cohen’s *d*: 1.1) with a power of >90% and significance level α < 0.05. LF, HF, and LF/HF ratio of heart rate were compared between groups and to baseline using non-parametric tests due to the reduced sample size. The data are represented as mean ± SEM and statistical significance was considered at a level of *P* < 0.05. All statistical analyses were performed using SPSS software version 20.0 (IBM SPSS Inc., Chicago, IL, United States). Graphs were plotted using GraphPad Prism 6.0 (GraphPad Software, La Jolla, CA, United States).

## Results

### Leptin Receptor Blockade Did Not Exacerbate Obesity

**FIGURE 1:**
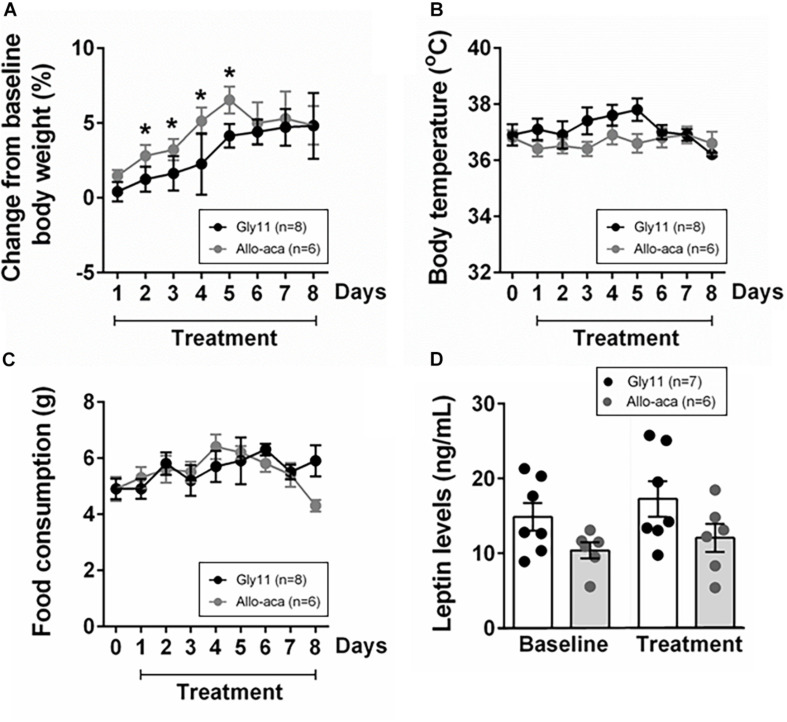
Leptin receptor blocker Allo-aca did not affect **(A)** body weight, **(B)** body temperature, **(C)** food consumption, and **(D)** plasmatic levels of leptin in NZO mice. **(A)** Lines represent the percentage of change in the body weight throughout the 8 days of treatment compared to baseline. **(B,C)** Line graphs represent the comparisons to baseline (day 0) and among the 8 days of treatment with either Allo-aca or control peptide Gly11. Mean values ± standard errors are shown. Mixed regression model. *Significant increase in body weight until day 5 of treatment in both groups (*P* < 0.05).

At baseline, both groups were obese, showing similar body weights (mean ± SE of 45.6 ± 0.6 g in Gly11 vs. 42.4 ± 1.7 g in Allo-aca, *P* > 0.05). Significant increases in body weight from baseline were observed until day 5 of treatment with either Allo-aca or control peptide (Figure 1A), suggesting no exacerbation of obesity with the leptin receptor blockade. Body temperature and food consumption before and after the treatment were also similar between the groups (Figures 1B,C). Leptin levels were slightly higher in the controls (14.9 ± 1.5 ng/ml) compared to the Allo-aca group (10.4 ± 1.7 ng/mL) at baseline, but this difference did not reach statistical significance (Figure 1D). Treatment with Allo-aca and control peptide did not affect plasma leptin levels.

### Leptin Receptor Blockade Attenuated Hypertension, but Did Not Affect Cardiac Autonomic Balance

**FIGURE 2:**
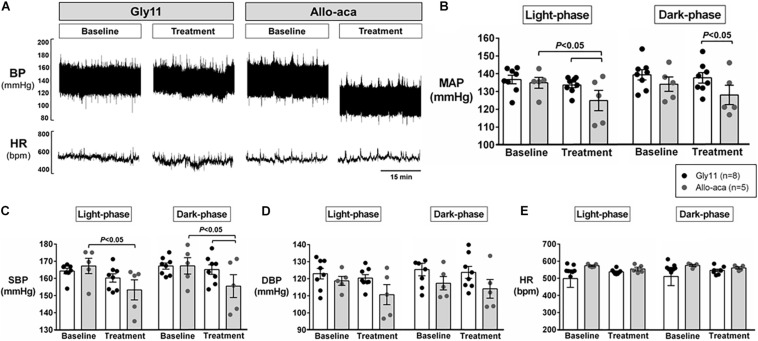
Systemic blockade of leptin receptor with Allo-aca attenuated hypertension in NZO mice. **(A)** Representative trace of blood pressure (BP) and heart rate (HR) recordings during light-phase at baseline and after the treatment with Allo-aca and control peptide. **(B)** Mean arterial pressure (MAP) significantly decreased with Allo-aca treatment compared to baseline and to control peptide Gly11 during light-phase. Blockade of leptin receptors reduced **(C)** systolic blood pressure (SBP) during light and dark-phase, but did not change **(D)** diastolic blood pressure (DBP) and **(E)** heart rate (HR). Graphs shown as averaged 24-h BP/HR ± standard errors. Mixed regression model.

**FIGURE 3:**
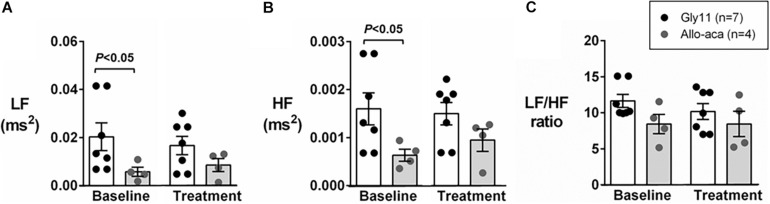
Leptin receptor blocker Allo-aca did not affect sympathetic and parasympathetic activities, and cardiac autonomic balance. **(A)** Low frequency (LF) and **(B)** high frequency (HF) components of heart rate were lower at baseline in Allo-aca group, but no significant effects of the treatment were observed. **(C)** LF/HF ratio was similar between groups and compared to baseline. Mean values ± standard errors are shown. Mann–Whitney U test and Wilcoxon signed-rank test.

Figure 2A shows a representative trace of blood pressure and heart rate recordings during light-phase at baseline and after the treatment with Allo-aca and control peptide. All NZO mice were hypertensive at baseline (Figure 2). Allo-aca effectively reduced the MAP during light-phase from 134.9 ± 3.1 to 124.9 ± 5.7 mmHg (*P* < 0.05) whereas the negative control peptide had no effect (Figures 2A,B). Leptin receptor blockade induced a smaller reduction in MAP during dark-phase, which was not significantly different compared to baseline, but it was lower than controls (127.9 ± 5.5 vs. 137.5 ± 3.0 mmHg, *P* < 0.01) (Figures 2A,B). The effects of Allo-aca treatment on MAP during dark-phase were not statistically significant when mixed-effects models were utilized to account for baseline differences in MAP between individual animals. Mice treated with Allo-aca exhibited a significant decrease in SBP during both light and dark-phases (Figure 2C) (*P* < 0.05). DBP was not significantly affected (Figure 2D). Heart rate was similar between the groups at baseline and was not affected by either Allo-aca or Gly11 (Figure 2E). Heart rate variability is shown in Figure 3. At baseline, LF and HF components of heart rate were lower in Allo-aca group compared to controls (Figures 3A,B) (*P* < 0.05). However, no significant effects of the treatment with either Allo-aca or control peptide were observed on LF and HF. LF/HF ratio was similar between groups and remained unchanged after treatment (Figure 3C), suggesting that the cardiac autonomic balance was not affected.

### Leptin Receptor Blockade Did Not Change Minute Ventilation or HVR

**FIGURE 4:**
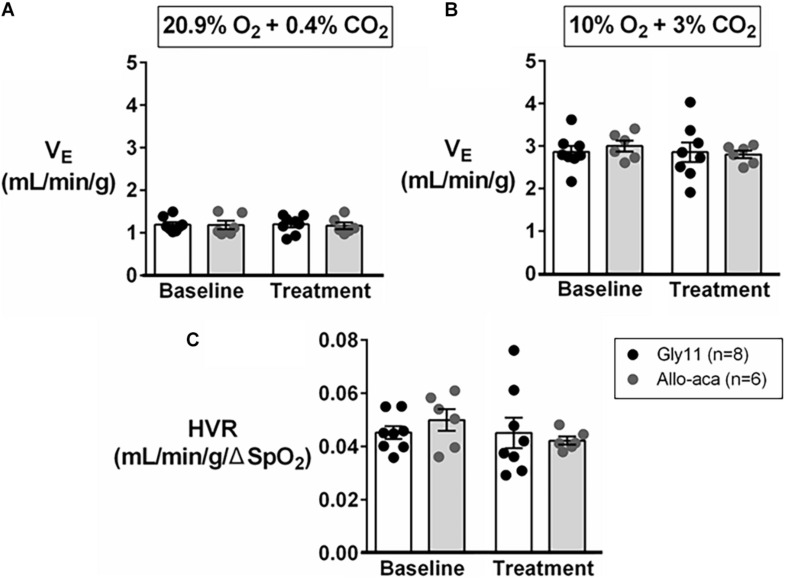
Hypoxic ventilation was not affected by the blockade of leptin receptors with Allo-aca. Allo-aca and control peptide Gly11 did not change minute ventilation (V_E_) at **(A)** normoxia (20.9% O_2_, 0.4% CO_2_) and **(B)** hypoxia (10% O_2_, 3% CO_2_), and **(C)** the hypoxic ventilatory response (HVR). Data were normalized by body weight. SpO_2_, oxygen saturation. Mixed regression model.

Normalized V_E_ during normoxia (1.2 ± 0.1 mL/min/g in both groups) and hypoxia (Gly11 2.9 ± 0.2 mL/min/g vs. Allo-aca 3.0 ± 0.1 mL/min/g) were similar at baseline in both groups (Figures 4A,B). Allo-aca and Gly11 did not change ventilation at 21% and 10% FiO_2_ (Figures 4A,B). Consequently, leptin receptor blockade did not affect HVR (baseline 0.05 ± 0.0 mL/min/g/% vs. Allo-aca 0.04 ± 0.0 mL/min/g/%) (Figure 4C).

### Leptin Receptor Blockade Did Not Exacerbate SDB

**FIGURE 5:**
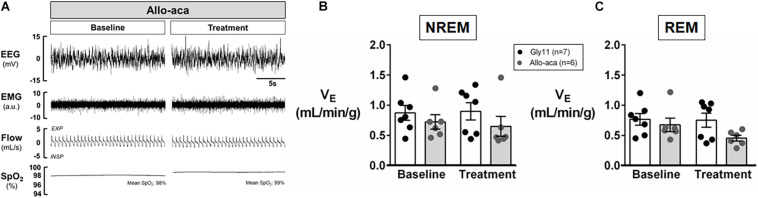
Leptin receptor blocker Allo-aca did not exacerbate sleep-disordered breathing in NZO mice. **(A)** Representative trace of NREM sleep at baseline and after the treatment with leptin receptor blocker Allo-aca. Minute ventilation (V_E_) was not significantly affected with either Allo-aca or control peptide compared to baseline during **(B)** NREM and **(C)** REM sleep. Mice treated with Allo-aca had a decreased V_E_ during REM sleep than controls after treatment, but no significant interaction was observed. Data were normalized by body weight. Mean values ± standard errors are shown. Mixed regression model.

Allo-aca and Gly11 had no significant effects on sleep architecture and both groups had similar total sleep time, sleep efficiency, and duration of NREM and REM sleep (Table 1). Leptin receptor blockade did not affect ventilation during sleep as represented in Figure 5A. Normalized V_E_ during NREM was similar between groups at baseline and remained unchanged after the treatment with Allo-aca and control peptide (Figure 5B). A slight decrease in V_E_ during REM sleep was observed in Allo-aca group, but it was not significantly different compared to baseline and to controls (Figure 5C). V_T_, RR, V_I_max, MIFR, and inspiratory DC remained unchanged with both Allo-aca and control peptide (Table 2). The systemic blockade of leptin receptors did not exacerbate SDB in NZO mice, indicated by no significant changes in the frequency of IFL breaths, apnea index, and ODI (Table 2).

**TABLE 2:** Ventilation during NREM and REM sleep in NZO mice at baseline and after the treatment with leptin receptor blocker Allo-aca or control peptide Gly11.

		Gly11 (*n* = 7)		Allo-aca (*n* = 6)	
		Baseline	Treatment	Baseline	Treatment
**NREM**	V_T_ (mL)	0.3 ± 0.0	0.4 ± 0.0	0.3 ± 0.1	0.3 ± 0.1
	RR (bpm)	107.5 ± 5.9	111.6 ± 5.0	95.9 ± 6.4	90.7 ± 5.4*
	V_I_max (mL/S)	3.1 ± 0.3	3.1 ± 0.3	2.9 ± 0.5	2.1 ± 0.4
	MIFR (mL/s)	2.0 ± 0.3	2.0 ± 0.3	1.8 ± 0.3	1.3 ± 0.3
	Inspiratory DC	0.3 ± 0.0	0.3 ± 0.0	0.3 ± 0.0	0.3 ± 0.0
	IFL (%)	4.8 ± 1.3	8.0 ± 2.3	4.4 ± 1.4	7.5 ± 2.5
	Mean SpO_2_ (%)	95.7 ± 0.7	95.6 ± 0.8	97.0 ± 0.5	97.0 ± 0.5
**REM**	V_T_ (mL)	0.3 ± 0.0	0.3 ± 0.0	0.3 ± 0.0	0.2 ± 0.0
	RR (bpm)	122.7 ± 5.3	123.6 ± 5.0	118.3 ± 5.8	116.1 ± 5.4
	V_I_max (mL/s)	2.8 ± 0.4	2.7 ± 0.3	2.5 ± 0.4	1.8 ± 0.3
	MIFR (mL/s)	1.6 ± 0.2	1.6 ± 0.2	1.5 ± 0.2	1.1 ± 0.2
	Inspiratory DC	0.4 ± 0.0	0.4 ± 0.0	0.3 ± 0.0	0.3 ± 0.0
	IFL (%)	18.3 ± 3.6	23.7 ± 4.7	16.6 ± 3.9	19.3 ± 5.1
	Mean SpO_2_ (%)	92.3 ± 1.0	92.1 ± 1.0	94.5 ± 1.0	95.2 ± 1.0
	Apneas (events/h)	31.4 ± 5.4	26.7 ± 5.3	31.8 ± 5.6	33.7 ± 5.1
	ODI (events/h)	8.4 ± 1.5	7.0 ± 1.4	6.2 ± 1.5	7.4 ± 1.4

Mean values ± standard errors are shown. V_*T*_, tidal volume; RR, respiratory rate; V_*I*_max, maximum inspiratory flow; MIFR, mean inspiratory flow rate; DC, duty cycle; IFL, inspiratory flow limitation; SpO_2_, oxyhemoglobin saturation; ODI, oxygen desaturation index. Mixed regression model. *P < 0.05 compared to control group.

## Discussion

Taken together, systemic blockade of leptin receptors attenuated hypertension without exacerbating obesity or SDB in NZO mice. To the best of our knowledge, this is the first study to suggest that obesity-related hypertension in NZO mice is, at least in part, related to leptin signaling. We showed that hypertensive NZO mice treated with leptin receptor blocker Allo-aca had significant reduction in blood pressure with no changes in cardiac autonomic balance. These effects were most pronounced during the light-phase when mice were more likely to sleep. Our data also suggest that leptin receptor blockade did not exacerbate obesity and obesity-induced SDB in NZO mice. First, NZO mice treated with leptin receptor blocker Allo-aca did not show changes in body weight and food consumption. Second, Allo-aca did not affect ventilation during hypoxia. Third, leptin receptor blockade in NZO mice did not alter ventilation, oxygen saturation, and the apnea rate during NREM and REM sleep.

### Role of Leptin in Obesity-Induced Hypertension

It is well known that leptin plays a key role in the pathogenesis of obesity-induced hypertension. Leptin activates the sympathetic nervous system and elevates blood pressure in a dose-dependent manner (Haynes et al., 1997a; Rahmouni et al., 2005a; Asferg et al., 2010; Shankar and Xiao, 2010; Machleidt et al., 2013; Shin et al., 2019a, 2021). Systemic blockade of leptin receptors abolishes hypertension in murine models of obesity and hyperleptinemia (Huby et al., 2016). Here, we used NZO mice to analyze the role of leptin receptor blockade on blood pressure. NZO mice develop hypertension at early age and the weight gain is accompanied by the rise in blood pressure (Ortlepp et al., 2000; Marchesi et al., 2009). However, the mechanisms underlying the development of obesity-related hypertension in NZO mice remain unknown. Polymorphism in leptin receptors has been identified in these animals (Igel et al., 1997; Kluge et al., 2000; Joost and Schürmann, 2014). Similar polymorphism is observed in New Zealand Black (NZB) mice (Igel et al., 1997), a strain that shares a common origin with NZO mice and develops high blood pressure despite the lack of obesity (Ortlepp et al., 2000; Marchesi et al., 2009). This evidence may implicate leptin signaling in the pathogenesis of hypertension in NZO mice. Our present study adds to this body of knowledge by demonstrating that hypertension in NZO mice is significantly attenuated by the systemic blockade of leptin receptors. Additionally, our findings suggest that the systemic blockade of leptin receptors did not change cardiac autonomic balance. In part, the small sample size and large variability among the mice could have accounted for the different sympathetic and parasympathetic activities at baseline between groups.

### Role of Leptin in Control of Breathing and SDB

Leptin is a potent stimulator of breathing. Systemic replacement of leptin in leptin-deficient *ob/ob* mice normalizes breathing and the hypercapnic ventilatory response (O’Donnell et al., 1999; Pho et al., 2016). Leptin infusions augment V_E_ under normoxic and hypoxic conditions, and increase HVR in lean rodents (Yuan et al., 2018; Caballero-Eraso et al., 2019). In rats, the effects of leptin on ventilation are blunted with the increase in leptin levels induced by high-fat diet, suggesting a compromised ventilatory adaptation possibly induced by the development of leptin resistance in obesity (Ribeiro et al., 2018). Our study was the first to analyze ventilation in NZO mice. Compared to our previous study, NZO mice showed lower levels of V_E_ and a lower HVR than lean C57BL/6J mice (∼3.6 mL/min/g), but similar ventilatory responses to hypoxia to leptin receptor-deficient *db/db* mice (HVR: 2.8 mL/min/g) (Caballero-Eraso et al., 2019). The blockade of leptin signaling did not affect HVR and ventilation during sleep in NZO mice, which could have two alternative explanations: (1) a floor effect of leptin on breathing in already hypoventilating NZO mice or (2) relatively modest elevations of leptin levels may not be sufficient to affect the ventilatory control in this strain. In our previous study, leptin infusion promoted a ∼30-fold increase in leptin levels in lean C57BL/6J mice (from undetectable to 34.9 ± 4.3 ng/mL) and this massive increase in leptin levels lead to changes in breathing (Caballero-Eraso et al., 2019).

### Potential Sites and Mechanisms of Leptin Action

Leptin regulates the metabolism and stimulates breathing acting on leptin receptors in the hypothalamus and medulla (Halaas et al., 1995; O’Donnell et al., 1999; Spiegelman and Flier, 2001; Friedman, 2009; Pho et al., 2016; Yao et al., 2016). Respiratory sites of leptin have been localized to the nucleus tractus solitarius (NTS) (Inyushkina et al., 2010; Yao et al., 2016; Do et al., 2020), retrotrapezoid nucleus/parafacial respiratory group (Bassi et al., 2014), and dorsomedial hypothalamus (Pho et al., 2021). In contrast, the sites of hypertensive effects of leptin have not been determined. Earlier studies have suggested that the central regulation of blood pressure by leptin is localized to the dorsomedial hypothalamus (Simonds et al., 2014; Gruber et al., 2021). Here, we showed that the systemic blockade of leptin receptors in NZO mice reduced blood pressure, but did not affect ventilation and metabolism, since food intake remained unchanged. Although Allo-aca initially induced weight gain, this effect was transient suggesting the progression of leptin resistance.

Our current data are consistent with an established paradigm that obese humans and rodents are resistant to beneficial metabolic and respiratory effects of leptin, but sensitive to detrimental hypertensive effects of this hormone (Maffei et al., 1995; Considine et al., 1996; Ip et al., 2000; Phipps et al., 2002; Yee et al., 2006; Fleury Curado et al., 2018; Berger et al., 2019). Leptin resistance in NZO mice has been attributed, at least in part, to an inadequate transport of leptin to the CNS (Halaas et al., 1995; Igel et al., 1997), while leptin receptor signaling in the CNS remained intact (Igel et al., 1997; Rizk et al., 1998; Hileman et al., 2002). Although Allo-aca penetrates the BBB (Otvos et al., 2011b), the dichotomy between the absence of central metabolic and respiratory effects of this leptin receptor blocker and the presence of hypotensive effects suggest that leptin may act peripherally to induce hypertension.

Peripheral sites, outside the BBB, have been implicated in the regulation of blood pressure and/or control of breathing, such as the carotid bodies. The carotid bodies are the main peripheral sensors of blood gases and pH located bilaterally at the bifurcation of the common carotid arteries (Ortega-Sáenz and López-Barneo, 2020). The denervation or resection of the carotid bodies effectively reduce blood pressure and HVR (Olson et al., 1988; Fletcher et al., 1992; McBryde et al., 2013; Fudim et al., 2015; Del Rio et al., 2016; Caballero-Eraso et al., 2019; Shin et al., 2019a). Our group has shown that leptin acts in the leptin receptors in the carotid bodies to induce hypertension and to stimulate breathing and HVR (Caballero-Eraso et al., 2019; Shin et al., 2019a). These effects are mediated through the activation of transient receptor potential melastatin 7 (TRPM7) channel and the blockade of TRPM7 channels abolishes the leptin-induced hypertension (Shin et al., 2019a, 2021). In the present study, the reduction in blood pressure of NZO mice with Allo-aca could be attributed to the peripheral blockade of leptin receptors in the carotid bodies, inhibiting the leptin-TRPM7 axis. Other peripheral sites could also be involved, including the blockade of leptin receptors in adrenal medulla (Haynes et al., 1997b; Huby et al., 2016). However, the blockade of leptin receptors in the current study did not affect the HVR, which may suggest that either the carotid bodies do not play a main role in the regulation of breathing or the peripheral control of breathing is mediated by other molecular mechanisms in NZO mice. Previous studies have suggested the role of protein tyrosine phosphatase 1b (Ptp1b), a negative regulator of leptin and insulin signaling, in the regulation of blood pressure (Belin de Chantemèle et al., 2009; Huby et al., 2016). Ptp1b knockout mice have increased leptin sensitivity despite the lack of obesity (Belin de Chantemèle et al., 2009; Huby et al., 2016). The treatment with Allo-aca in Ptp1b knockout mice treated endothelial dysfunction and hypertension, but its effects on ventilation remain unclear (Huby et al., 2016).

### Limitations

Our study had several limitations. First, we used systemic blockade of leptin receptor in NZO mice and therefore we were unable to distinguish between peripheral and central effects of leptin and to localize the sites of leptin action on blood pressure. Additional techniques to block leptin signaling, such as intracerebroventricular injections of leptin receptor blockers, virus-induced gene silencing at the particular sites or the treatment with leptin antibodies, which do not penetrate BBB, are necessary to confirm our findings. Second, we had a relatively small sample size to analyze the effects of Allo-aca. However, even with a reduced number of animals, our effect sizes were relatively large enabling us to detect the statistical differences in blood pressure. Third, we treated NZO mice acutely with Allo-aca. Chronic protocols could show more long-term beneficial effects of leptin receptor blockade on blood pressure and/or highlight the detrimental effects of leptin inhibition on obesity and SDB. Fourth, blood samples were obtained after a period of 6 h of food deprivation for sleep studies, which could have accounted for the modest hyperleptinemia observed in our NZO mice (∼10–17 ng/mL). Finally, our therapeutic regimen for Allo-aca could explain the more pronounced hypotensive effects of leptin receptor blockade during light-phase. Our protocol also did not allow us to estimate the onset of hypotensive effects of Allo-aca throughout the 8 days of treatment.

## Conclusion

In conclusion, systemic leptin receptor blockade attenuates hypertension in NZO mice without exacerbating obesity and SDB. These findings support that leptin is a key regulator of blood pressure and highlight the potential applicability of pharmacological blockade of leptin signaling as a therapy for patients with obesity-induced hypertension.

## Data Availability Statement

The raw data supporting the conclusions of this article will be made available by the authors, without undue reservation.

## Ethics Statement

The animal study was reviewed and approved by the Johns Hopkins University Animal Care and Use Committee (ACUC).

## Author Contributions

LK, LO, and VP: conceived and designed the study. LK, MKS, and HP: data collection. LK, MKS, HP, LO, LP, and VP: data analyses and interpretation. LK, LP, and VP: drafting the manuscript. LK, MKS, HP, LO, MA, ST, LP, and VP: critical revision of the manuscript. All authors contributed to the article and approved the submitted version.

## Conflict of Interest

LO was employed by the company Arrevus Inc. and OLPE LLC. The remaining authors declare that the research was conducted in the absence of any commercial or financial relationships that could be construed as a potential conflict of interest.
